# Efficient Azo Dye Biodecolorization System Using Lignin-Co-Cultured White-Rot Fungus

**DOI:** 10.3390/jof9010091

**Published:** 2023-01-07

**Authors:** Su Sun, Pengyang Liu, Mati Ullah

**Affiliations:** 1 College of Urban Construction, Wuchang Shouyi University, Wuhan 430064, China; 2 Key Laboratory of Molecular Biophysics of MOE, Department of Biotechnology, College of Life Science and Technology, Huazhong University of Science and Technology, Wuhan 430074, China

**Keywords:** white-rot fungus, alkali lignin, azo dye, dye decolorization, co-culture

## Abstract

The extensive use of azo dyes by the global textile industry induces significant environmental and human health hazards, which makes efficient remediation crucial but also challenging. Improving dye removal efficiency will benefit the development of bioremediation techniques for textile effluents. In this study, an efficient system for azo dye (Direct Red 5B, DR5B) biodecolorization is reported, which uses the white-rot fungus *Ganoderma lucidum* EN2 and alkali lignin. This study suggests that the decolorization of DR5B could be effectively enhanced (from 40.34% to 95.16%) within 48 h in the presence of alkali lignin. The dye adsorption test further confirmed that the alkali-lignin-enhanced decolorization of DR5B was essentially due to biodegradation rather than physical adsorption, evaluating the role of alkali lignin in the dye biodegradation system. Moreover, the gas chromatography/mass spectrometry analysis and DR5B decolorization experiments also indicated that alkali lignin carried an excellent potential for promoting dye decolorization and displayed a significant role in improving the activity of lignin-modifying enzymes. This was mainly because of the laccase–mediator system, which was established by the induced laccase activity and lignin-derived small aromatic compounds.

## 1. Introduction

Water is an essential component for the survival of human beings and all organisms. However, with the rapid development of industrialization, growing water pollution is becoming one of the major challenges the industrialized world faces [1,2,3]. The production of colorants has been estimated to exceed 7 × 10^5^ tons annually worldwide, and approx. 10–15% of dyes are directly discharged into the aquatic environment, presenting one of the main sources of pollution across the world [4,5,6]. Azo dyes are important synthetic dyes for commercial applications as a result of their chemical stability and versatility [7,8,9]. Nevertheless, they also seriously threaten living organisms because of their toxicity and potential carcinogenicity [10,11,12]. Therefore, in the face of the pollution of azo dyes in the environment, more efficient methods for removing azo dyes are expected.

White-rot fungi, a group of classical lignocellulolytic microbes, have been widely used for the treatment of dyes in industrial wastewater because of their outstanding non-specific degradation capability for an extensive range of substrates [13,14,15,16]. The nonspecific ligninolytic enzymes such as laccase, manganese peroxidase (MnP), lignin peroxidase (LiP), and universal peroxidase (VP) play a crucial role in biological decolorization due to their action on different types of substrates [14,17,18,19]. In general, LiP, MnP, and VP have high redox potentials and require the use of H_2_O_2_ as an additive to initiate radical reactions. On the other hand, laccases have relatively low redox potentials, and thus the free radical reactions can be initiated only by providing molecular oxygen as an electron acceptor; consequently, their use is more convenient than other peroxidases [20,21].

It has been reported that the degradation of refractory pollutants could be enhanced in a simulated natural environmental system containing lignocellulosic wastes or lignocellulose-derived compounds [19,22,23]. Furthermore, we previously showed that water-soluble phenolic extract from lignocellulosic wastes could enhance the production of laccase and improve dye decolorization [24,25]. However, many researchers mainly attributed such an enhancement to the sugars released from lignocellulose or to the advantages of solid fermentation systems for fungal growth and enzyme expression [26,27,28,29]. A number of studies suggested that the addition of artificial aromatic compounds could increase the degradation efficiency of xenobiotics [30,31,32,33]. However, few studies have characterized the significance of lignin waste for dye degradation. Lignin is a complex biopolymer, with phenylpropane as its structural unit, that typically constitutes 15–40 wt.% (dry) of lignocellulose in plant materials (or biomass) [34,35,36,37,38,39]. In addition, lignin is considered a waste by-product of biorefinery and the kraft-pulping process of lignocellulose. The white-rot fungi are well known for their depolymerization and valorization of lignin into aromatic compounds [40]. Meanwhile, dyes and lignin have similar structures, which all rely on the same extracellular lignolytic degradation enzyme system of white-rot fungi to realize their own degradation. Therefore, there is likely to be a certain connection between the metabolic processes of lignin and dyes by white-rot fungi [16,41,42]. To better understand the effect of lignin (when it is used as a co-substrate) on dye degradation by white-rot fungi, a comprehensive study was carried out to identify the potential and effective use of alkali lignin in the decolorization of DR5B by the white-rot fungus *Ganoderma lucidum* EN2.

## 2. Materials and Methods

### 2.1. Microorganism and Chemicals

The fungal strain *G. lucidum* EN2 was obtained from rotting wood in the Shennongjia Nature Reserve, Hubei Province, China, while the research was conducted only in the laboratory, did not involve endangered or protected species, and had no biosecurity concerns. The strain was incubated at 4 °C in a potato dextrose agar (PDA) slope medium before use.

Direct red 5B (DR5B), a sulfonated azo dye used widely in the textile industry, was purchased from Colorfran S.A. (Monterey, Mexico), while sodium azide was bought from Aldrich (Milwaukee, WI, USA). Glucose and mineral salts were acquired from Sinopharm Chemical Reagent Co., Ltd (Shanghai, China). Alkali lignin (CAS No. 8068-05-1) and the low-molecular lignin-derived compounds were obtained from Sigma-Aldrich (St. Louis, MO, USA).

### 2.2. Decolorization Experiments with Lignin

The *G. lucidum* EN2 was incubated (for 5 days at 28 °C) in 250 mL Erlenmeyer flasks containing 100 mL of modified Kirk medium [43] with a speed of 150 rpm. A 10.0% (*v/v*) pre-cultured fungus was then introduced into the decolorization medium, formed by the addition of 175 mg L^−1^ of DR5B to the modified Kirk medium, and cultivated for 6 days.

The influence of alkali lignin on the decolorization of DR5B was investigated by adding different amounts of alkali lignin (0, 0.15, 0.30, and 0.60 g L^−1^) to the afore-mentioned dye decolorization medium. The filtrate obtained from the dye and lignin-free fermented group was labeled “DR5B”, the one from the alkali lignin pre-cultured fermented group was labeled “Lignin”, the one acquired from the blending of both DR5B and the alkali lignin was labeled “DR5B+Lignin”, while that from the no dye and lignin fermented group was labeled “Blank”. The supernatants were collected periodically and their dye decolorization effects, pHs, and enzymatic activities were measured. The pH values of the samples were measured with a Phs-2C apparatus (Shanghai Sanxin Instrument Factory, Shanghai, China), and all the experiments in the study were performed in triplicate.

### 2.3. Dye Adsorption Assay

The physical adsorption rate of DR5B by alkali lignin was measured via the ultraviolet–visible (UV/VIS) spectrophotometer (Thermo Fisher Scientific Inc., Waltham, MA, USA) at the maximum absorption wavelength of DR5B (510 nm).

The dye adsorption capacity of fungal mycelia was measured after the recuperation of the adsorbed dye via extraction with 70% (*v/v*) methanol during a 24 h incubation at room temperature of the mycelia collected from the culture medium via centrifugation at 8000 rpm for 10 min [44].

### 2.4. Effect of Cell-Free Extracellular Liquid on Dye Decolorization

Crude cell-free extracellular liquids (CFEL) of *G. lucidum* EN2 from the above-mentioned different treatment conditions were obtained by filtering it through a 0.22 µm filter flask after 72 h of cultivation. Then, a sterilized 25 mg L^−1^ (final concentration) DR5B was added to the crude extracellular liquids, placed at 28 °C, and the OD values were recorded at different incubation times. A total of 10 mM of NaN_3_ was also added to each extracellular liquid as an enzyme inhibitor to investigate the effect of laccase on DR5B decolorization.

### 2.5. Metabolites Analysis Using Gas Chromatography/Mass Spectrometry

The identification of dye and lignin metabolites was conducted using gas chromatography/mass spectrometry (GC/MS). After DR5B decolorization for 96 h, the decolorized medium was harvested and centrifugated at 5000 rpm for 20 min to gather the supernatant and draw out the metabolites with a uniform volume of ethyl acetate. The GC/MS analysis of metabolites was executed on an Agilent 6890 GS/5973 MS (Agilent Technologies, Inc., Bethlehem, PA, USA) fitted with a DB-wax capillary column (30 m × 0.25 mm internal diameter; 0.25 mm film thickness). An aliquot of 1 µL of the sample was administered into the GC/MS apparatus, and helium was used as a carrier gas at a flow rate of 1 mL min^−1^. The column temperature program was 40 °C for 1 min followed by 10 °C min^−1^, providing the final heating rate up to 250 °C, and maintaining the time hold for 15 min.

### 2.6. Effect of the Lignin-Derived Compound on DR5B Decolorization and Laccase Induction

Lignin-derived compounds (including guaiacol, phenol, 4-vinyl guaiacol, benzaldehyde, methoxybenzene, and ethyl guaiacol) were dissolved in ethanol, filter sterilized, and added to the dye decolorization systems containing *G. lucidum* EN2 or laccase (at a final concentration of 5–100 µM). Two control groups (with and without the addition of ethanol to the fungal culture) were established, and the supernatants were taken out for the measurement of the laccase enzyme activity and to analyze the decolorization of the azo dye.

### 2.7. Analytical Methods

The decolorization of DR5B was measured in the culture filtrates and spectrophotometrically monitored at the wavelength of maximum adsorption (510 nm). The decolorization rate (%) was evaluated according to the formula P = (A1 − A2)/A1 × 100%, where A1 and A2 signify the initial and final absorbance units, respectively.

The crude enzyme (laccase) was collected by salting out the *G. lucidum* EN2 supernatant with ammonium sulfate. After that, the crude laccase was purified using hydrophobic interaction chromatography and ion-exchange chromatography [45]. For measuring laccase activity, ABTS was used as a substrate, while the supernatant was mixed with 10 mM of ABTS and 100 mM of a sodium acetate buffer (pH 4.5) [46]. The MnP activity was determined by mixing the supernatant with 1mM of a MnSO_4_ solution, 100 mM of a malonic acid buffer (pH 4.5), and 0.1 mM of H_2_O_2_ [47]. For the LiP assay, 4 mM of veratryl alcohol, 100 mM of a sodium tartrate buffer (pH 3.0), 5 mM of MnSO_4_, and 1 mM of H_2_O_2_ were mixed with the supernatant [48]. All assay mixtures were composed of 1 mL of a buffer solution, 1 mL of ABTS, and 1 mL of the supernatant. The mixture change in the adsorption was measured at a wavelength of 420 nm for laccase, 270 nm for MnP, and 310 nm for LiP. One enzyme activity unit (1U) was defined as the absorbance that increased or decreased by 0.001 min^−1^.

All the data were collected in triplicate and expressed as means (SD), whereas the graphical analysis was conducted using ORIGIN 9.0 (Microcal Software, Northampton, MA, USA). All statistical tests were performed using the IBM SPSS Statistics 21 software package. In all the tests, the level of statistical significance was assumed to be at *p* < 0.05.

## 3. Results and Discussion

### 3.1. Effect of Alkali Lignin on Dye Decolorization

**Figure 1:**
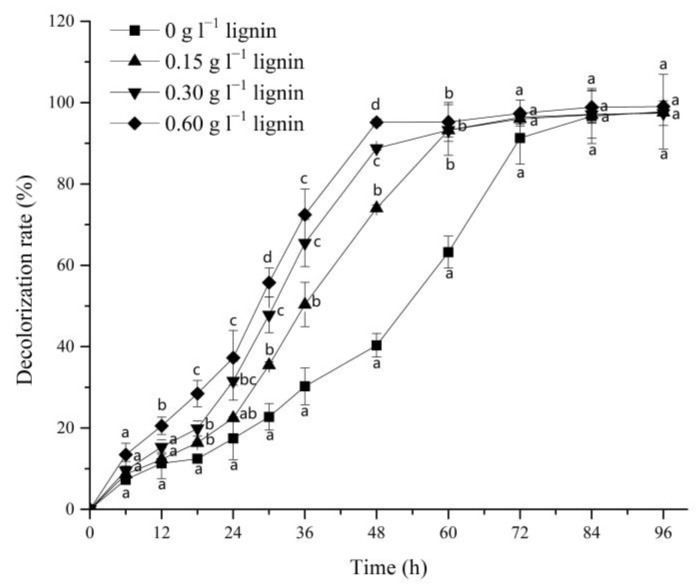
Decolorization rate of Direct Red 5B (DR5B) by *G. lucidum* EN2 in the presence of different contents of alkali lignin (0, 0.15, 0.30, and 0.60 g L^−1^). The vertical line on each bar indicates the standard deviation for three replicates (SD, *n* = 3). Different letters represent significant differences between treatment groups at each time point, as determined with two-way ANOVA followed by LSD or Tukey multiple comparison tests (*p* < 0.05).

The effect of alkali lignin and the decolorization efficiency of *G. lucidum* EN2 on azo dye DR5B was monitored by adding different amounts of alkali lignin to the dye decolorization medium. The DR5B removal percentage by *G. lucidum* EN2 was significantly increased by the introduction of alkali lignin, and this acceleration showed a positive correlation with the increasing dosage of alkali lignin. These results showed that the decolorization rates of the 3 experimental groups could increase up to 74.12%, 89.23%, and 95.16% with alkali lignin (i.e., 0.15, 0.30, and 0.60 g L^−1^, respectively) within 48 h (Figure 1). However, only 40.34% decolorization was achieved in the control group without alkali lignin. The most significant differences in the DR5B decolorization percentages compared with the group without lignin were observed at 48 h. Before 72 h, the decolorization rates of the three experimental groups still exceeded that of the control group. These results specified that alkali lignin could enhance the decolorization of azo dye DR5B by *G. lucidum* EN2, which is consistent with previous studies using lignocellulosic waste for textile dye bioremediation. This result is also supported by a previous study, in which the consumption of lignocellulosic wastes as a co-substrate considerably increased the azo dye decolorization efficiency of *Bjerkandera* sp. BOL 13 [49]. Similarly, another study conducted in vivo also reported an enhancement in the azo dye bio-decolorization by *Echinodontium taxodii* when co-cultured with lignin [15].

### 3.2. Physical Adsorption of DR5B by Alkali Lignin and Fungal Mycelia

**Figure 2:**
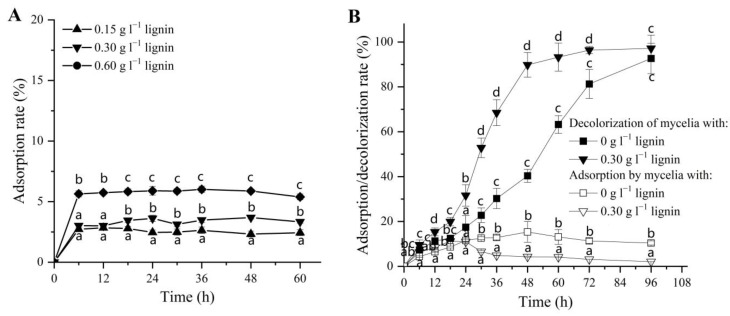
Physical adsorption of DR5B by alkali lignin and fungal mycelia. (**A**) Physical adsorption rate of DR5B when it was incubated with alkali lignin at contents of 0.15, 0.30, and 0.60 g L^−1^. (**B**) Physical adsorption rate of DR5B by *G. lucidum* EN2 mycelia in the presence of 0 and 0.30 g L^−1^ alkali lignin. Decolorization by *G. lucidum* EN2 mycelia of DR5B in the presence of 0 and 0.30 g L^−1^ alkali lignin. The vertical line on each bar indicates the standard deviation for three replicates (SD, *n* = 3). Different letters represent significant differences between treatment groups at each time point, as determined with two-way ANOVA followed by LSD or Tukey multiple comparison tests (*p* < 0.05).

The mycelium adsorption capacities for dyes were previously reported in many studies [50], some of which could also relate to the increased adsorption capacity in the presence of alkali lignin [51]. Therefore, it was necessary to study the effect of alkali lignin and fungal mycelia on the physical adsorption of DR5B. Initially, the DR5B was incubated with the alkali lignin to study its effect on the physical adsorption of dye DR5B. The maximum adsorption percentage (<6%) was achieved within 6 h when the highest alkali lignin content (0.60 g L^−1^) was added, and there was no significant increase or decrease thereafter (Figure 2A). In contrast, the dye decolorization rate reached 95.2% at 60 h after adding 0.6g/L of lignin, which was significantly different from the highest decolorization rate (<6%) caused by lignin adsorption (Figure S1). This dye adsorption assay showed that the increased dye decolorization efficiency with the alkali lignin addition during DR5B biodegradation was not mainly caused by the physical adsorption of alkali lignin.

Furthermore, the physical adsorption ability of *G. lucidum* EN2 mycelia for the dye DR5B was also investigated in both experimental conditions (i.e., in the presence and absence of alkali lignin) during the biodegradation process (Figure 2B). The adsorption and total decolorization of dyes were very comparable during the first 18 h, suggesting that the initial dye decolorization by the fungus was indeed mainly the result of adsorption during that period. In the presence of alkali lignin, *G. lucidum* EN2 was able to decolorize up to 65.48% of the azo dye within 36 h (0.30 g L^−1^), followed by an increase of up to 88.78% within 48 h (Figure 2B). However, the physical adsorption of dye by the mycelium remained at a relatively stable level after 36 h (approx. 2%) when compared with the control group (approx. 10%) (Figure 2B). This suggests that physical adsorption had no significant effect on the total decolorization during the whole DR5B removal process. These results clearly showed that the enhanced dye degradation rate after the addition of alkali lignin was mainly the result of the biodegradation of *G. lucidum* EN2 when compared with the physical adsorption by alkali lignin or mycelium.

### 3.3. Effect of Alkali Lignin on Laccase Activities during the Degradation of DR5B by G. lucidum EN2

**Figure 3:**
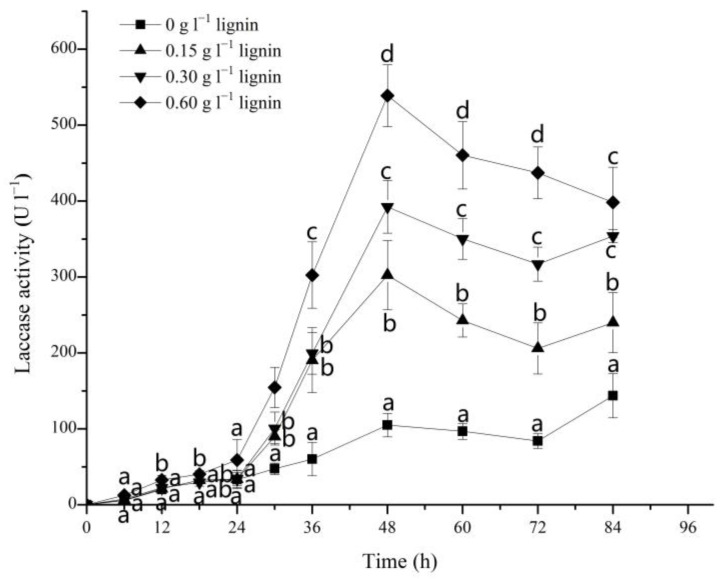
Laccase activities during the DR5B decolorization process by *G. lucidum* EN2 in the presence of different contents of alkali lignin (0, 0.15, 0.30, and 0.60 g L^−1^). The vertical line on each bar indicates the standard deviation for three replicates (SD, *n* = 3). Different letters represent significant differences between treatment groups at each time point, as determined with two-way ANOVA followed by LSD or Tukey multiple comparison tests (*p* < 0.05).

Ligninolytic enzymes, such as laccase, LiP, MnP, and VP, mainly secreted by white-rot fungi, can effectively degrade lignin and other persistent organic pollutants [52,53]. In this study, laccase was the only ligninolytic enzyme that could be detected in the utilized dye degradation system with *G. lucidum* EN2. As visible from Figure 3, the laccase enzyme activity was only 105.00 U L^−1^ in the absence of alkali lignin after the incubation of *G. lucidum* EN2 for 48 h in DR5B. By increasing the lignin content from 0 to 0.15, 0.30, and 0.60 g L^−1^, the laccase activity was significantly increased from 105.00 to 302.50, 392.50, and 539.00 U L^−1^ at 48 h, respectively. This increase in the extracellular laccase activity was about 2.5–4 times that of the control group and was thus consistent with a previous study which also reported a similar increase in the laccase activity by using various lignocellulose residues [54].

At a later stage of the lignin addition system, laccase activity also decreased and was accompanied by the complete decolorization of DR5B at 72 h. The rapid increase in the dye decolorization rates (74.12, 89.23, and 95.16% with different contents of alkali lignin added at 48 h) followed a similar trend to the variation in laccase activity. The elevation in laccase activity and the synthesis of various aromatic compounds by white-rot fungi from lignin degradation have also been reported in many previous studies [55,56]. Yang et al. [57] in 2013 reported that lignin model compounds and other related lignin derivatives increase the activity of ligninolytic enzymes in fungi. The result showed that the addition of alkali lignin (or compounds released from degraded alkali lignin) may stimulate laccase activity in *G. lucidum* EN2, and the augmented laccase activity may also deliver a significant contribution to the bio-decolorization of DR5B.

### 3.4. Effect of Alkali Lignin Degradation of Extracellular Liquid on DR5B Removal

To identify whether the growing laccase induced by alkali lignin was the main reason for the effective decolorization, another DR5B decolorization experiment with cell-free extracellular liquid was performed.

**Figure 4:**
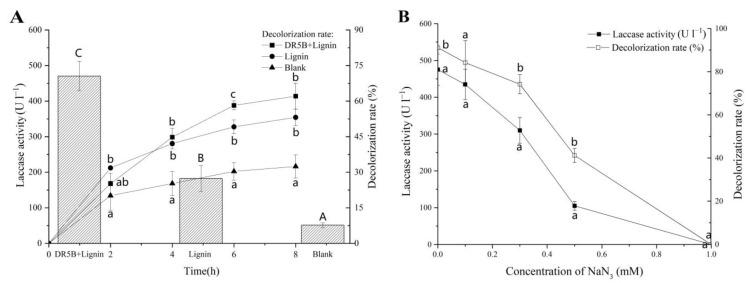
Effect of cell-free extracellular liquid (CFEL) and inhibition of laccase on DR5B decolorization. (**A**) Decolorization of DR5B (25 mg L^−1^) by the CFEL extracted from *G. lucidum* EN2 incubated with or without lignin and DR5B for 48 h. “DR5B+Lignin”: CFEL obtained from *G. lucidum* EN2 incubated with lignin and DR5B and added Kirk medium; “Lignin”: CFEL extracted from *G. lucidum* EN2 fermented with lignin and added Kirk medium; “Blank”: CFEL extracted from *G. lucidum* EN2 fermented with Kirk medium only; (**B**) Inhibition of laccase and decreased decolorization with increasing sodium azide concentration. The vertical line on each bar indicates the standard deviation for three replicates (SD, *n* = 3). Different letters represent significant differences between treatment groups at each time point, as determined with a two-way ANOVA followed by LSD or Tukey multiple comparison tests (*p* < 0.05).

DR5B (final concentration 25 mg L^−1^) was incubated with cell-free extracellular liquid harvested from different fermentation culture media (including the groups “Lignin+DR5B”, “Lignin”, and “blank”). As shown in Figure 4A, the laccase activities in the groups Lignin+DR5B and Lignin were significantly higher than that in the Blank group. The DR5B decolorization assay showed that the lignin-containing degradation samples of extracellular liquid (62.1% in the “Lignin+DR5B” group and 55.1% in the “Lignin” group) resulted in remarkable decolorization compared with the Blank group (32.4%) after 8 h of incubation (Figure 4A). The lignin co-substrate promote the laccase activity of *G. lucidum* EN2, and the lignin-containing degradation of extracellular liquid significantly promoted the decolorization of DR5B (Figure 3 and Figure 4A).

To further determine the role of laccase in DR5B decolorization, different concentrations of the laccase-specific inhibitor NaN_3_ were added to the extracellular liquid of each group, and the resulting abilities for dye removal were tested. Laccase activity decreased with an increasing concentration of NaN_3_. Meanwhile, the DR5B decolorization ability was reduced with a decrease in laccase activity. As the concentration of NaN_3_ reached 1 mM, the laccase activity of extracellular fluid was completely suppressed and no more decolorization of DR5B could be observed (Figure 4B). Therefore, this result strongly indicated that laccase plays a prominent role in the degradation of DR5B, and the addition of alkali lignin increased the laccase activity, which may be one method to promote the dye decolorization rate. This importance of laccase could also relate to many previously reported studies [58,59] and is also consistent with our recently reported study, which showed that the improvement of laccase activity increased the dye decolorization efficiently [60].

### 3.5. Characterization of Metabolites Resulting from Degradation of Both DR5B and Alkali Lignin by G. lucidum EN2

**Figure 5:**
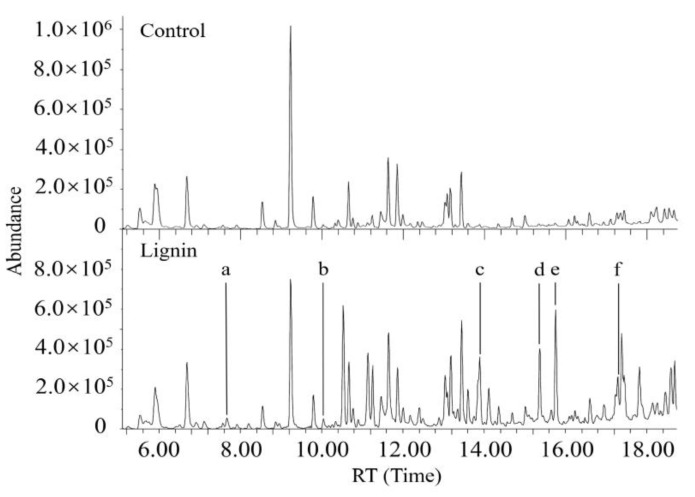
Total ion chromatogram of the compounds extracted from dye degradation in the absence (up) or presence (down) of alkali lignin. a-f show the peaks of chemicals present in the lignin culture. a: methoxybenzene (RT 7.7); b: benzaldehyde (RT 10.0); c: guaiacol (RT 13.9); d: phenol (RT 15.4); e: 4-vinyl guaiacol (RT 15.7); f: ethyl guaiacol (RT 17.2).

The increased laccase activity with the alkali lignin addition was demonstrated using enzymatic assays. However, insoluble lignin is not accessible and, thus, cannot induce enzyme activity; moreover, it also cannot regulate dye degradation [61,62]. Considerable research has shown that many low-molecular aromatic compounds derived from lignin can increase the enzyme activity of laccase in fungal systems and can also act as mediators of lignin-degrading enzymes to accelerate the degradation of dyes [15,63,64]. The comparative GC/MS analysis results further confirmed the degradation of alkali lignin and identified the released aromatic compounds during the degradation of alkali lignin by *G. lucidum* EN2. Ethyl-acetate-extraction samples obtained from the supernatant of DR5B fermentation by *G. lucidum* EN2 containing alkali lignin were compared with the lignin-free samples. However, it was difficult to capture the trace and transient amounts of lignin metabolic intermediates throughout the short-term decolorization. Several low molecular weight aromatic compounds (LMCs), such as methoxybenzene (RT 7.7), benzaldehyde (RT 10.0), guaiacol (RT 13.9), phenol (RT 15.4), 4-vinyl guaiacol (RT 15.7) and ethyl guaiacol (RT 17.2), were still captured and detected from the sample with alkali lignin (Figure 5). These recovered low aromatic compounds have been identified as lignin degradation intermediates, which strongly suggests that alkali lignin was degraded by *G. lucidum* EN2 accompanied by the azo dye degradation process. Additionally, several of these lignin-derived aromatic compounds, including phenols and guaiacols, are considered potential laccase substrates and may also act as natural and proficient laccase mediators for the oxidation of xenobiotics including numerous kinds of dyes [24,33,65,66,67]. In the laccase–mediator system, aromatic compounds could be oxidized into stable radicals by enzymes and act as redox mediators to oxidize other compounds. Hence, the lignin metabolic intermediates were further evaluated for dye decolorization.

### 3.6. Effect of Different Lignin-Derived Aromatic Compounds on Dye Decolorization and Laccase Activity

**Figure 6:**
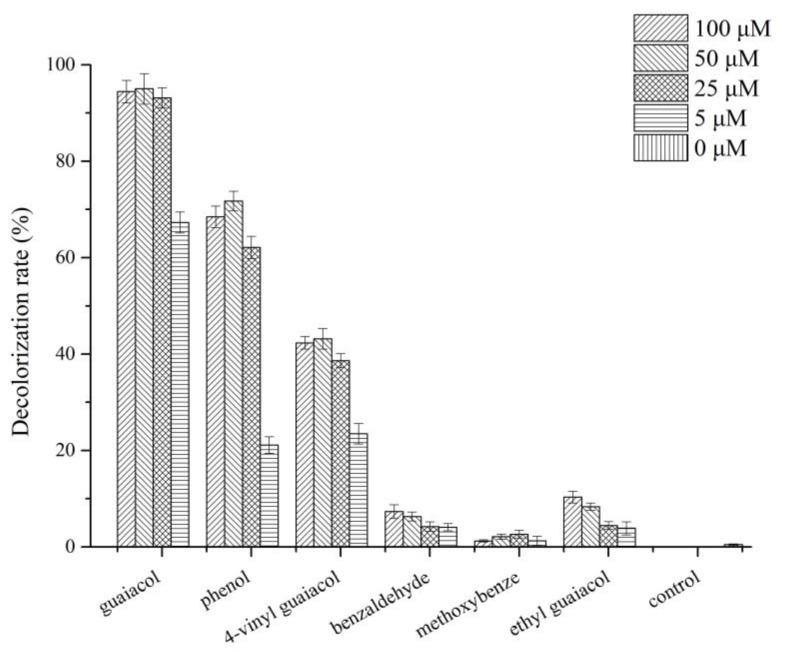
Evaluating lignin-derived compounds based on decolorization of DR5B within 1 h. Decolorization of DR5B (100 mg L^−1^) within 1 h with purified laccase (final concentration: 1 U mL^−1^) and intermediators (0, 5, 25, 50, and 100 µM).

Laccase present a promising role in the degradation of azo dye DR5B according to the presented results (Figure 4). The laccase from *G. lucidum* EN2 cultures was purified by fractionation with ammonium sulfate, followed by hydrophobic interaction and ion-exchange chromatography for further purification (Figure S2). Six lignin-derived model compounds (including guaiacol, phenol, 4-vinyl guaiacol, benzaldehyde, methoxybenzene, and ethyl guaiacol) were selected, and their effects on the decolorization of azo dyes were examined with the purified laccase (Figure 6). The purified laccase slowly decolorized DR5B in the absence of the aforementioned screened lignin metabolite by 3% after 2 h of incubation. However, the decolorization of DR5B could be accelerated with the presence of lignin-derived compounds including guaiacol (2-methyloxy phenol), 4-vinyl guaiacol (4-vinyl-2-methoxy phenol), phenol, ethyl guaiacol (4-ethyl-2-methoxy phenol), and benzaldehyde at different concentrations (Figure 6). Among these, guaiacol achieved the highest reaction and decolorization rate (>90%) within 1 h at 25 µM. Decolorization of DR5B was also significantly enhanced by different concentrations of phenol (21.12, 62.13, 71.72, and 68.47%) and 4-vinyl guaiacol (23.49, 38.62, 43.17, and 42.32%) (i.e., 5, 25, 50, and 100 µM) (Figure 6). The same phenomenon of DR5B decolorization was also observed in the screened aromatic compounds added to the *G. lucidum* EN2 degradation systems. This result was consistent with an earlier study where phenol-type derivatives demonstrated a significant induction effect on laccase expression in many species of white-rot fungi [60,68,69,70]. Additionally, for laccase oxidation reactions, these low-molecular-weight phenol derivatives could also act as natural mediators in the degradation of polycyclic aromatic hydrocarbons by laccase–mediator systems [15,33,70].

The degradation of lignin may yield numerous aromatic compounds [71,72]. However, the lower quantity of lignin intermediates made these relatively difficult to detect because of the low amount of lignin addition, existing transients, and the interference of its aromatic compound secretion by the white-rot fungi. Nevertheless, parts of the lignin-degraded compounds were still captured. Several phenolic mediators tested here were also verified to enhance the removal of DR5B as well as mediate laccase to accelerate the azo dye decolorization. Based on the aforementioned results, the addition of alkali lignin promoted the DR5B decolorization by *G. lucidum* EN2 through two pathways: firstly, the addition of alkali lignin enhanced the laccase activity by inducing laccase expression to promote dye decolorization; secondly, a number of low molecular weight compounds derived from lignin degradation served as mediators and increased the dye decolorization efficiency in the *G. lucidum* EN2 system.

## 4. Conclusions

This study shows the potential of lignin in promoting the decolorization of the azo dye DR5B and enhancing the activity of lignin-modifying enzymes. The practice of using alkali lignin resulted in the efficient removal of DR5B from water by *G. lucidum* EN2. The adsorption experiments showed that the enhanced decolorization of DR5B was primarily through biodegradation rather than the physical adsorption of lignin or mycelia. Laccase played a major role in DR5B decolorization. Moreover, if added to the proposed dye biodegradation system, alkali lignin could improve DR5B decolorization through two pathways. Firstly, alkali lignin could be degraded into small molecular aromatic compounds, which increased laccase activity to promote the DR5B decolorization; secondly, several of the small molecular aromatic compounds, especially phenol and guaiacol, acted as mediators of laccase and, thus, significantly enhanced the dye degradation by *G. lucidum* EN2. These findings not only provide new insights into the underlying mechanisms of significantly enhanced dye decolorization in the presence of alkali lignin, but they will also profoundly impact the development of sustainable solutions for the effective environmental remediation of azo dyes.

## Data Availability

Not applicable.

## Supplementary Materials

The following supporting information can be downloaded at https://www.mdpi.com/article/10.3390/jof9010091/s1, Figure S1: The comparison of DR5B adsorption and the DR5B decolorization of *G. lucidum* EN2 in the presence of different contents of alkali lignin (0.15, 0.30, and 0.60 g L^−1^) at 60 h. Figure S2: SDS-PAGE analyses of purified laccase of *G. lucidum* EN2. Lane 1, purified laccase; Lane 2, protein molecular mass marker.
