# Supplementary material for: Efficient Azo Dye Biodecolorization System Using Lignin-Co-Cultured White-Rot Fungus
Source: J Fungi (Basel). 2023 Jan 7;9(1):91. doi: 10.3390/jof9010091 (PMC9866751; doi:10.3390/jof9010091)
Supplement: Supplementary file 1 [file jof-09-00091-s001.zip › jof-2099673-supplementary.pdf]

Supplementary Materials

Efficient Azo Dye Biodecolorization System Using Lignin Co-Cultured White-Rot Fungus

Su Sun [1,2,*], Pengyang Liu [2] and Mati Ullah [2]

[1] College of Urban Construction, Wuchang Shouyi University, Wuhan 430064, China

[2] Key Laboratory of Molecular Biophysics of MOE, Department of Biotechnology, College of Life Science and Technology, Huazhong University of Science and Technology, Wuhan 430074, China

[*] Correspondence: sunsu@hust.edu.cn

Supplementary Figure S1

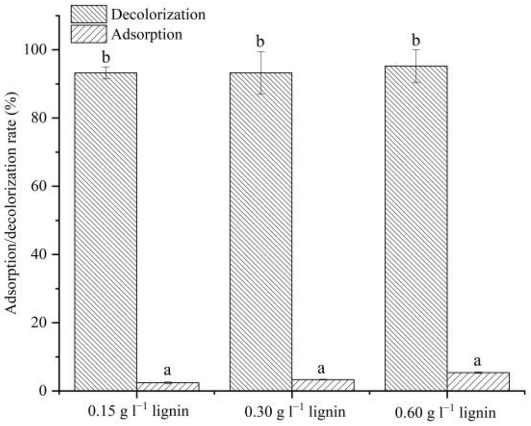

Figure S1. The comparison of DR5B adsorption and the DR5B decolorization of *G. lucidium* EN2 in the presence of different contents of alkali lignin (0.15, 0.30, and 0.60 g l^{-1}) at 60 h. The vertical line on each bar indicates the standard deviation for three replicates (SD, n = 3). Different letters represent significant differences between treatment groups at each time point, as determined by two-way ANOVA followed by LSD or Tukey multiple comparison test (p < 0.05)

Supplementary Figure S2

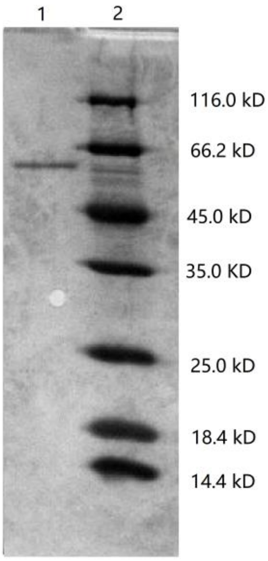

Figure S2. SDS-PAGE analyses of purified laccase of *G. lucidum* EN2. Lane 1: purified laccase; Lane 2: protein molecular mass marker.
